# Missed appendicitis after self-induced abortion

**Published:** 2011-11-13

**Authors:** Damien Punguyire, Victor Kenneth Iserson

**Affiliations:** 1 Kintampo Municipal Hospital, Kintampo, Ghana; 2 Department of Emergency Medicine, The University of Arizona, 4930 N. Calle Faja, Tucson, AZ, USA

**Keywords:** Appendicitis, misdiagnosis, abortion, pregnancy, misoprostol

## Abstract

Female lower abdominal pain poses diagnostic difficulties for clinicians, especially when little more than the history and physical examination are available. A girl presented with constant lower abdominal pain after taking misoprostol for pregnancy termination. She was eventually referred to a rural District Hospital, where a laparotomy demonstrated acute appendicitis. After treating herself for a self-diagnosed pregnancy with illegally provided misoprostol, this patient presented with persistent lower abdominal pain. The differential diagnosis included ectopic pregnancy and all other causes of female abdominal pain. Yet diagnosing two diseases in the same anatomical area at the same time contradicts diagnostic parsimony. System problems in resource-poor areas can limit access to healthcare services and encourage dispensing potentially dangerous medications without clinicians’ authorization. It is dangerous to rely on patients’ self-diagnoses while neglecting other diagnoses. More than one diagnosis may be needed to explain temporally and anatomically related symptoms.

## Background

Female lower abdominal pain has long posed diagnostic difficulties for clinicians, especially when they have had little more than the history and physical examination to guide them. Even first-trimester pregnancy diagnoses were expensive and time consuming until disposable qualitative immunological β-HCG assays became widely available in the 1980s. More accurate diagnoses have resulted from using transabdominal and transvaginal ultrasonography, computerized tomography, and diagnostic laparoscopy. These modalities, however, usually are not available in resource-poor areas.

When they are lacking, as in the case presented here, clinicians may continue to follow the wrong diagnostic path, leading to unnecessary surgical procedures and potentially devastating treatment delays.

## Patient and case report

A 19-year-old sexually active high school student with a history of regular menses believed she was pregnant after missing her period for three months. Still in school and not ready to have a child, she wanted to terminate the pregnancy but was reluctant to visit a healthcare professional. She could not pay for such a visit and even though she had government-sponsored medical insurance, it did not cover abortion services. Her girlfriend, the only person in whom she confided, suggested misoprostol for termination; she had never been pregnant or previously used misoprostol. A Licensed Chemical Seller (LCS) sold her misoprostol without a prescription.

The fourth of 6 siblings, the patient had been sent to school from a rural farming community and lived near the school with her two younger sisters. Her first coitus was at 16 years, but she had never used birth control or condoms, claiming that she did not believe that she would get pregnant or contract sexually transmitted diseases (STDs). Her religious beliefs did not influence her behaviour.

Vaginal bleeding began a few hours after taking 200 mcg misoprostol orally and the same dose vaginally. She bled for four days, accompanied by severe constant lower abdominal pain. Nevertheless, she remained at home. Five days later, the bleeding subsided but right sided pelvic pain persisted. Two days later, she went to a hospital near her home, where she was given the clinical diagnosis of septic incomplete abortion with severe anaemia (Hgb 7.3 g/dl). They had no ultrasound or transfusion capability. Twenty-four hours after performing a manual vacuum aspiration (MVA) with no observable products of conception, they transferred the patient with a diagnosis of pelvic abscess. Despite her level of illness, she came to our district hospital, about 20 minutes away by taxi, the common transport for ambulatory patients.

On arrival, the thin and small-for-her-age woman looked pale and obviously ill. Her vital signs were temperature 37.8°C, BP 100/60 mm/Hg, pulse 100/min, regular and good volume. Her abdomen was diffusely tender with peritoneal signs. Since the one surgeon for the facility was operating, she was sent to the maternity ward, where the nurses, immediately recognizing her level of illness, started an IV, drew labs, and notified the surgeon.

She was found to have a Hgb 6.1 g/dl, WBC 15.9 x109/L, with 10.8% lymphocytes, 9.0% mononuclear cells, and 80.2% neutrophils. A physician-performed ultrasound transabdominal examination showed an empty but bulky uterus with fluid in the dependant parts of the abdominal cavity. The presumptive diagnosis was pelvic abscess, although other processes were considered that also required surgery. She received two units of whole blood, additional crystalloids, ciprofloxacin and metronidazole.

**Figure 1:**
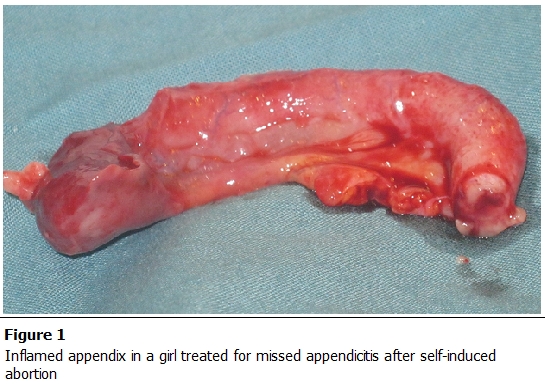
Inflamed appendix in a girl treated for missed appendicitis after self-induced abortion

Under general anesthesia, exploration through a Pfannenstiel incision demonstrated that the uterus, fallopian tubes and both ovaries were normal, but that the peritoneal cavity contained a large quantity of serosanguinous fluid. The incision was extended in an inverted “T” to the epigastrium for a more extensive laparotomy. After draining the fluid, an inflamed appendix was found adhering to the cecum and no other pathology was identified. An appendectomy was performed (Figure 1), the peritoneal cavity was lavaged and the abdomen was closed primarily.

### Post-operative course

Post-operative management included prolonged use of parenteral antibiotics. During the post-operative healthcare education, the patient stated that she did not intend to use either contraception or STD protection in the future, relying instead on sexual abstinence.

## Discussion

### Presentation

This patient presented to a relatively resource-poor hospital after treating herself for a self-diagnosed pregnancy. Although she never took a pregnancy test, her self-diagnosis was reasonable given her menstrual and sexual history. Doing a pregnancy test at the initial hospital would have been positive if she were still pregnant or had recently aborted. The persistent right lower quadrant abdominal pain should, however, have steered those clinicians in the direction of immediate referral, rather than first subjecting the patient to an MVA and then delaying her transfer by 24 hours. While lower abdominal pain, usually colicky, is often associated with misoprostol use, the cramps would have dissipated long before this patient presented to the hospital.

### Misoprostol

Misoprostol is a prostaglandin analogue with uterotonic properties. It is 94% to 98% effective in inducing abortions when used orally, vaginally, or both. The failure rate is greater if used after 9 weeks gestation [1,2]. The drug commonly causes vaginal bleeding within 12 hours of administration, with vaginal bleeding and uterine cramps being the most common reasons for women to seek medical care[3]. In this case, misoprostol use may have complicated and delayed appropriate treatment.

Under Ghanaian law, however, the patient should never have obtained misoprostol without first seeing an experienced physician. Misoprostol is a class C medication that may only be prescribed by doctors at a District Hospital or higher level institution, and only dispensed by registered pharmacists. In this case, an LCS illegally provided her with misoprostol. LCS's usually have only a few weeks of training and staff small retail outlets that sell over-the-counter medication. The LCS could be fined or their outlet closed down for selling misoprostol, although it is unclear whether this occurred.

#### Diagnostic Process

The differential diagnosis of persistent abdominal pain, especially following menstrual irregularities in a 19-year-old woman, has the potentially life-threatening ectopic pregnancy near the top of the list. It should also include all other causes of abdominal pain in an adult woman, including acute appendicitis. Acute appendicitis is the most common general surgical problem encountered during pregnancy [4]. The incidence averages 1 in 550 pregnancies [5]. With a recently pregnant patient, the potential dangers inherent in missing an ectopic pregnancy diagnosis would have been serious. It's unclear why that possibility wasn't considered, with the patient transferred immediately for diagnosis and treatment.

When the patient arrived at our hospital, her clinical picture plus free fluid on the ultrasound made it clear that she required surgery, probably for an ectopic pregnancy or, given her lack of STD protection, a pelvic abscess. Appendicitis was a lesser consideration but, as Hickam's Dictum tells us, patients can have as many diseases as they wish [6]. This contrasts with the more commonly used and cited principle of Ockam's Razor, which says that “of two explanations, each capable of explaining a given phenomenon, one should choose the simpler of the two” [7]. Or, in other words, the hoof beats you hear probably mean horses, not unicorns.

Even in non-pregnant women, however, it can be very difficult to differentiate appendicitis from pelvic inflammatory, gastrointestinal, and urinary tract disorders. Up to 1/3 of these patients are initially misdiagnosed. Such misdiagnosis is associated with an increased incidence of perforation and abscess formation, as well as prolonged hospitalization [8]. Differentiating appendicitis from gynecological disease often requires adequate ultrasound capability (the hospital had only transabdominal probes on a non-moveable machine) and laparoscopy (not available)[9].

One would imagine that this brush with death would alter the patient's view of safe sexual activity. Yet, when receiving education from nurses on these practices, this relatively well-educated patient demonstrated a cavalier attitude toward both birth control and sexually transmitted diseases. She asserted that she would rely on abstinence. Unfortunately, history shows that this will most likely lead to both pregnancy and sexually transmitted diseases.

## Conclusion

System problems in resource-poor areas can limit access to simple laboratory and healthcare services due to their unavailability in healthcare facilities or patients’ lack of healthcare education or poverty. One problem that is difficult to remedy is unsafe sexual practices, even among better educated young women.

In many countries, those selling medications often subvert official regulations and dispense potentially dangerous medications without a clinician's authorization. This leads to many problems, not the least of which is obscuring or delaying serious diagnoses and appropriate treatment.

Clinicians must be wary of relying on patients’ self-diagnoses while disregarding others that may better explain their symptomatology. The differential diagnosis for a woman's lower abdominal pain is extensive; neglecting other potential diagnoses risks increased morbidity (as in this case) or mortality. Sometimes clinicians must make more than one diagnosis to explain temporally and anatomically related symptoms.
